# Dopamine dysregulation syndrome after prescription of dopamine agonists: a case report

**DOI:** 10.1192/j.eurpsy.2022.1857

**Published:** 2022-09-01

**Authors:** M.L. Costa, A. Cerame

**Affiliations:** 1 Hospital Universitario Severo Ochoa, Psychiatry, Leganes, Spain; 2 Hospital Universitario José Germain, Hospital De Día, Leganes, Spain

**Keywords:** dopamine dysregulation syndrome, prevention of mental disorders, psychostimulants, addictive disorders

## Abstract

**Introduction:**

We present the case of a 65-year-old woman with multiple and chronic psychosomatic symptoms. Due to motor impairments she was diagnosed in 2009 with Parkinson’s Disease (PD) by the neurology department and empirical treatment with levodopa was prescribed. However, the patient increased her levodopa intake by three times the recommended dose. The patient presented many adverse effects, including psychotic symptoms, that were interrupted after the levodopa intake was ended during a two month internament in a psychiatric unit. Dopamine dysregulation syndrome (DDS) is a condition in which patients with PD increase their levodopa intake without an objective worsening of motor symptoms. Higher-than-prescribed doses are taken by patients who develop tolerance and dependence to dopaminergic agonists.

**Objectives:**

To analyse the prevalence of DDS, its diagnosis and treatment as well as the identification of risk factors.

**Methods:**

A case report is presented alongside a review of the relevant literature regarding DDS.

**Results:**

The available evidence suggests that the main risk factors for DDS are a history of mood disorders and behavioural disorders, but more studies are needed. Given that DDS is considered a rare adverse effect, physicians usually overlook voluntary dose increase by patients.

**Conclusions:**

DDS, even though uncommon, has severe adverse effects such as dependence and acute psychosis. Before prescription of dopamine agonists, individual risk factors (such as psychiatric comorbidities or history of substance abuse) should be assessed. Also, patients and families should be informed and trained in alarm signs detection. Further studies would be justified to determine DDS prevalence, early diagnosis and treatment.

**Disclosure:**

No significant relationships.

